# Assessing phase discrimination *via* the segmentation of an elemental energy dispersive X-ray spectroscopy map: a case study of Bi_2_Te_3_ and Bi_2_Te_2_S

**DOI:** 10.1039/c7ra08594j

**Published:** 2018-02-15

**Authors:** J. B. Byrnes, A. A. Gazder, S. Aminorroaya Yamini

**Affiliations:** Institute of Superconducting and Electronic Materials, University of Wollongong New South Wales 2500 Australia; Electron Microscopy Centre, University of Wollongong New South Wales 2500 Australia; Department of Engineering and Mathematics, Sheffield Hallam University Sheffield S1 1WB UK S.Aminorroaya@shu.ac.uk

## Abstract

The present case study critically assesses the efficacy of a previously proposed segmentation methodology as a means to discriminate phases *via* post-processing the image of an elemental map. In the Bi_2_Te_2.5_S_0.5_ multiphase compound, the reference spectra of the Bi_2_Te_3_ and Bi_2_Te_2_S phases are distinct enough to effectively distinguish two phases during map acquisition. Since the counts of the sulphur-K peak in the X-ray emission data are significantly higher for Bi_2_Te_2_S compared to Bi_2_Te_3_, the segmentation methodology exploits this variation and enables successful phase discrimination *via* post-processing the image of the elemental map.

## Abstract 2

The present case study critically assesses the efficacy of a previously proposed segmentation methodology as a means to discriminate phases *via* post-processing the image of an elemental map.

## Graphical abstract

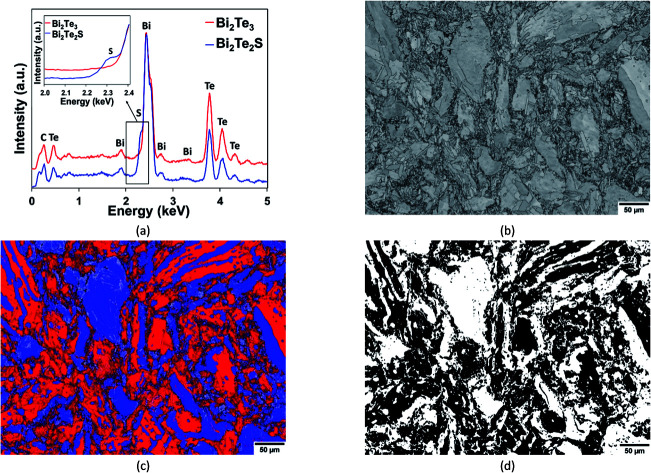

## 1. Introduction

Over the last decade, bismuth telluride based alloys have been investigated due to their exceptional performance as room temperature thermoelectric coolers and generators. Recent studies have shown that multiphase thermoelectric compounds exhibit higher conversion efficiencies than their single-phase counterparts.^1–4^ In the case of Bi–Te–S (stoichiometrically referred to as Bi_2_Te_2.5_S_0.5_), the alloy system comprises two phases; namely, Bi_2_Te_3_ and Bi_2_Te_2_S. Both phases have a trigonal crystal structure (space group 166, *R*3̄*m*) with the following lattice parameters: *a* = *b* = 4.39 Å, *c* = 30.47 Å (Bi_2_Te_3_), *a* = *b* = 4.18 Å, *c* = 29.45 Å (Bi_2_Te_2_S) with *α* = *β* = 90° and *γ* = 120°. Bi_2_Te_3_ consists of five layers in three blocks in the sequence [Te_2_–Bi–Te_1_–Bi–Te_2_]_0_ – [Te_2_–Bi–Te_1_Bi–Te_2_]_1/3_ – [Te_2_–Bi–Te_1_–Bi–Te_2_]_2/3_ with subscript fractions indicating the *z* translation of the blocks within the hexagonal unit cell.^5^ In the case of Bi_2_Te_2_S, the S atoms are substituted at the Te_1_ sites.^6^

To understand how the electronic transport properties of multiphase Bi_2_Te_2.5_S_0.5_ are influenced by the microstructure, micro-texture and fractions of individual phases, accurate phase discrimination is an essential first step. However, the above close correspondence in the crystal structure and lattice parameters of Bi_2_Te_3_ and Bi_2_Te_2_S renders phase discrimination *via* electron back-scattering diffraction patterns impossible.††As a general rule of thumb, effective phase discrimination using electron back-scattering diffraction patterns is only possible when individual phases have either distinct crystal structures or a minimum ∼10% difference in their lattice parameters.^20^

In this regard, energy dispersive X-ray spectroscopy (EDS) coupled with EBSD provides the appropriate solution. Here the user pre-defines reference spectra corresponding to the unique chemistries of each phase prior to mapping. Consequently, when the electron beam rasters across the sample surface during mapping, the characteristic X-rays emitted at each dwell point (based on a user-defined step size) first identify a phase based on its unique chemistry; following which the indexing of the electron back-scattering diffraction pattern is undertaken. In the case of Bi_2_Te_3_ and Bi_2_Te_2_S, their reference spectra are distinct enough to effectively discriminate the two phases during map acquisition.

In cases when reference spectra are not distinct enough to discriminate phases during map acquisition, post-processing of the EBSD map is undertaken to segment the phases. In the past, phase segmentation often relied on thresholding or multi-peak mathematical modelling of the frequency distribution of the band contrast (BC, or image/pattern quality, IQ, PQ) or band slope (BS) of the acquired electron backscattering patterns (EBSPs) or the size, aspect ratio, internal misorientation and boundary misorientation profiles of subgrains/grains.^7–10^ While these methods have been reviewed in detail in ref. 11, the main drawbacks of phase segmentation using EBSD-based parameters are summarised as follows. (i) Since each EBSD map tends to be uniquely based on microscope set-up and map acquisition parameters, it causes issues when delineating unique grayscale contrast ranges (in the case of BC/IQ/PQ/BS thresholding) for the various phases over multiple samples. (ii) Sub-dividing IQ/PQ/BC distributions based on multi-peak mathematical modelling is based on the assumption that each phase comprises symmetric, Gaussian sub-distributions and does not provide any means to account for overlaps between phases in the distributions.

Alternatively, a segmentation methodology was recently developed to discriminate granular bainite and bainitic ferrite (phases with crystal structure and lattice parameters that are very similar to alpha-iron) during the post-processing of a combined EDS + EBSD map of a transformation induced plasticity steel.^11^ The variation in carbon-K counts in the X-ray emission data between granular bainite and bainitic ferrite was exploited *via* a combination of: (i) grayscaling, (ii) image binarising, (iii) binary image inversion, (iv) local neighbourhood density thresholding and dilation of individual pixels and (v) median filtering; in order to effectively discriminate them.

In ref. 11 Monte-Carlo simulations of electron beam-sample interactions showed that even though carbon (a low density element at 2.267 gm cm^−3^) was used to segment the phases, the carbon X-ray emission data: (i) was obtained from localised volumes which were smaller than the smallest resolved substructures. (ii) did not suffer from plural scattering events arising from neighbouring phases and, (iii) did not contain channelling artefacts as anomalously high X-ray emissions and strong preferred orientations were absent.

While ref. 11 clearly showed that phase discrimination using low density elemental data is possible, no analyses of the absolute error associated with the segmentation methodology was undertaken. Thus, the present case study of Bi_2_Te_3_ and Bi_2_Te_2_S provides an ideal opportunity to critically assess the efficacy of the segmentation methodology as a means to discriminate phases. The segmentation methodology described can be applied to similar multiphase systems, where crystal structures of each phase are difficult to discern. For example, other thermoelectric systems such as multiphase quaternary lead chalcogenides and other novel bismuth telluride alloys could require phase segmentation *via* the proposed segmentation methodology.^12–16^ In this case, phase segmentation of Bi_2_Te_2.5_S_0.5_ is presented due to its promising thermoelectric properties.^17^ The counts of the sulphur-K peak in the X-ray emission data (similar to carbon, sulphur is also a low density element at 2.067 gm cm^−3^) are significantly higher for Bi_2_Te_2_S compared to Bi_2_Te_3_. Consequently, the segmentation methodology can be used to exploit the variation in the sulphur X-ray emission data and enable phase discrimination *via* post-processing the image of the elemental map. Comparing the phase maps obtained after map acquisition and segmentation on a per-pixel basis shows that when the segmentation parameters are optimised, the absolute error in phase discrimination *via* segmentation is ∼5%.

## 2. Experimental

### Materials synthesis and sample preparation

Polycrystalline Bi_2_Te_2.5_S_0.5_ was prepared by mixing stoichiometric quantities of high purity Bi (99.999%), Te (99.999%) and dried S (99.9%) in vacuum-sealed quartz ampoules at a residual pressure of ∼10^−4^ Torr. The sample was homogenised at 1123 K for 10 h and quenched in cold water followed by annealing at 673 K for 72 h.

The obtained ingots were hand-ground to powder with agate mortar and pestle. The powders were sintered into 12 mm diameter disc-shaped pellets using spark plasma sintering at 633 K and an axial pressure of 50 MPa for 300 s under vacuum. The sample for subsequent EBSD analysis was cut from the sintered disc such that the compression direction was normal to the surface. The sample surface was polished up to 1 μm diamond and then subjected to Ar-ion milling on a Leica EM RES101 at 4 kV for 1 h.

### Combined EDS + EBSD mapping

Combined EDS + EBSD mapping was undertaken for a 485.95 × 368.70 μm^2^ area in the centre of the Bi_2_Te_2.5_S_0.5_ surface using a JEOL JSM-7001F field emission gun – scanning electron microscope operating at 15 kV accelerating voltage and ∼5.5 nA probe current. X-ray emission spectra and electron back-scattering diffraction patterns were collected by the Oxford Instruments (OI) 80 mm^2^ X-Max EDS and Nordlys-S(II) EBSD detectors, respectively; both of whom interface with the Oxford Instruments Aztec software suite.

The EBSD mapping conditions were optimised prior to mapping by assigning 49 and 46 reflectors for the Bi_2_Te_3_ and Bi_2_Te_2_S phases respectively, 4 × 4 binning, 1 background frame, a Hough resolution of 60 while concurrently indexing up to 8 bands of individual electron back-scattering patterns *via* the Oxford Instruments “Refined Accuracy” algorithm. This algorithm improves the accuracy of Kikuchi band identification over conventional low-resolution 2D identification by correcting for the parabolic shape of Kikuchi bands on the phosphor screen of the detector. The obtained EBSD achieved an overall indexing rate of 94.37% with most zero solutions occurring at grain boundaries.

**Fig. 1:**
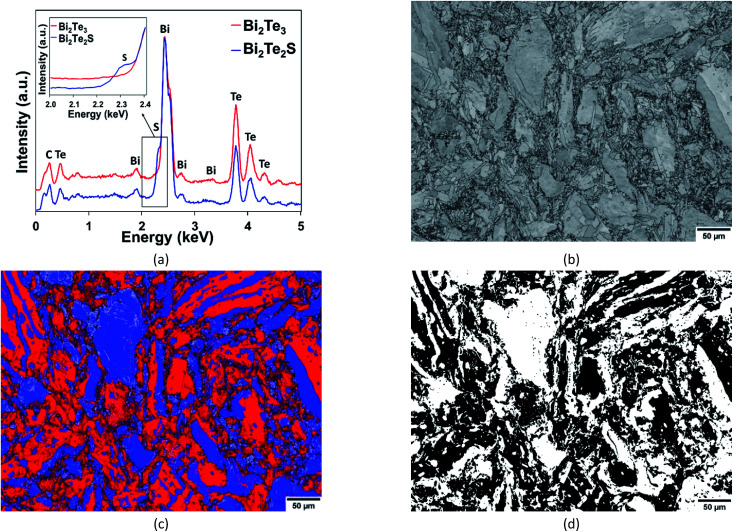
(a) Reference EDS spectra of Bi_2_Te_3_ (red) and Bi_2_Te_2_S (blue) obtained prior to mapping and used to distinguish the phases during combined EDS + EBSD mapping. (b) Band contrast and (c) phase distribution maps of Bi_2_Te_3_ (red) and Bi_2_Te_2_S (blue). (d) Binarised phase distribution map of Bi_2_Te_3_ (black) and Bi_2_Te_2_S (white). In (c), LAGBs = silver, HAGBs = black. The map in (d) is the reference phase map used to calculate the absolute error in the phase map obtained from segmentation (Fig. 2e) on a per-pixel basis.

A step size of ∼0.24 μm was equivalent to an EDS map resolution of 2040 × 1530 pixels. A 20 keV energy range, auto-selection of number of channels, a process time of 3 and detector dead time of ∼48–55% were used during mapping. Reference spectra corresponding to the unique chemistries of each phase were obtained prior to EDS + EBSD mapping (Fig. 1a). Sulphur is present in both phases due to its ease of diffusion at the temperatures imposed during sample fabrication. Over the full ‘TruMap’ area, the sulphur-K counts returned a Gaussian distribution (relative frequency *versus* counts per second (cps)) with the highest and maximum count rates of 1450 cps and 21 650 cps respectively. When multiplied by the mean dwell time of 0.0247 s per pixel, 36 and 534 counts were returned as the highest and maximum counts, respectively.

### Post-processing of the combined EDS + EBSD map

**Fig. 2:**
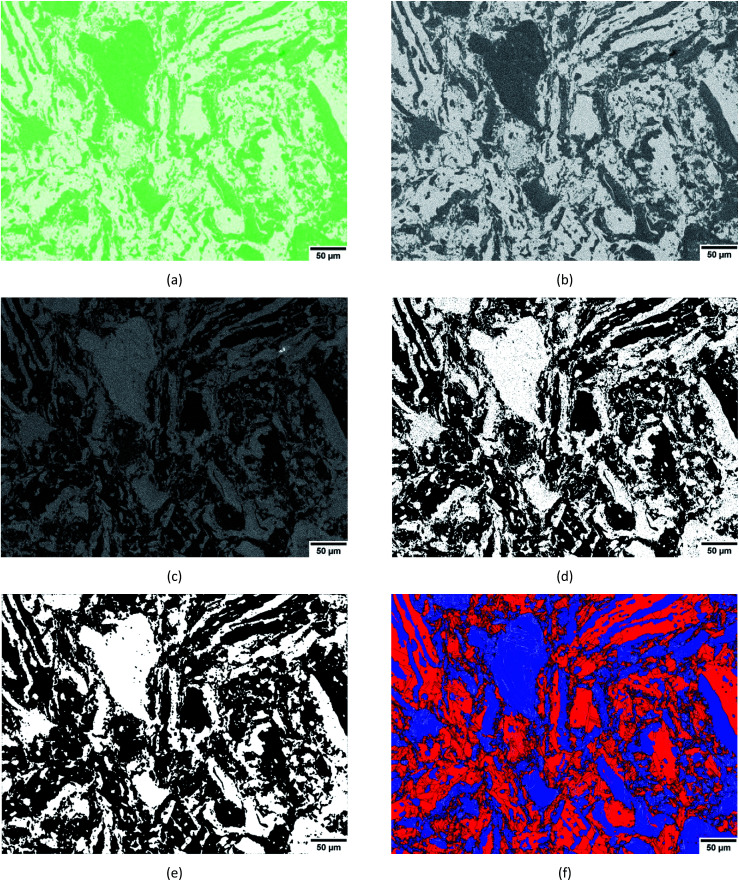
An example workflow for phase discrimination *via* segmentation. (a) The TIFF image of the map of relative distribution of sulphur-K counts imported into Gatan DigitalMicrograph. (b) Grayscaling of the image in (a). (c) Interactive thresholding between 200 and 255, binarising and inverting the contrast of the image in (b). (d) Applying a local neighbourhood density threshold of 1 white pixel in a 3 × 3 neighbourhood matrix of 8 first neighbour pixels to the image in (c). (e) Applying a median filter of size 3 to the image in (d). (f) Re-assigning white and black pixels as red (Bi_2_Te_3_) and blue (Bi_2_Te_2_S) for qualitative comparison with Fig. 1c. In (f), LAGBs = silver, HAGBs = black.

The EBSD map was cleaned using the OI Channel-5 software suite *via* methods described elsewhere.^11,13,18^ Wild orientation spikes were eliminated and zero solutions filled-in through cyclic extrapolation down to 5 neighbours. Herein, low-angle grain boundaries (LAGBs) are defined as misorientations between 2° to 15° and high-angle grain boundaries (HAGBs) as misorientations >15°. Subgrain reconstruction involved setting a minimum misorientation angle of 2° to improve the angular resolution limit and retain orientation contrast information. A constant minimum spatial resolution of 7 times the nominal step size was maintained to define the smallest substructure. The post-processed phase map (Fig. 1c) was binarised as shown in Fig. 1d. The latter figure was used to calculate the absolute error in the phase maps obtained by segmentation (Fig. 2). The segmentation methodology and subsequent absolute error analysis is detailed in the following sections.

### Phase discrimination *via* segmentation of the sulphur X-ray emission map

Fig. 2 is representative of an example workflow for phase discrimination *via* phase segmentation.‡‡The images shown in Fig. 2c and e were obtained using a neighbourhood matrix = 3 × 3, neighbourhood density threshold = 1, dilation kernel size = 0, median filter size = 3, as shown in Table 1. The sulphur-K map obtained in OI Channel-5 was exported as a TIFF image (Fig. 2a). Thereafter, the image was imported into Gatan DigitalMicrograph in order to undertake phase discrimination *via* the freeware scripts^19^ as follows (these scripts can be found at http://www.dmscripting.com/,^19^ and are hereafter referred to by their names as provided on the website). Grayscaling of the image was achieved by converting from RGB to a real image using the intensity of the green colour channel (Fig. 2b).

The “Interactive Thresholding” script was used to limit the grayscale threshold between 200 and 255. Thereafter, the image was binarised by assigning values of 0 and 1 to black and white pixels, respectively. The “Invert Image Contrast” script was then used to invert the binary image (Fig. 2c).

Following this, the “Local Neighbourhood Density Threshold and Dilation” script was invoked. In this step, a local neighbourhood surrounding a pixel of interest (placed in the centre of a square grid) is defined as a 3 × 3 matrix of 8 first neighbour pixels, 5 × 5 matrix of 8 + 16 = 24 first and second neighbour pixels or a 7 × 7 matrix of 8 + 16 + 24 = 48 first, second and third neighbour pixels and so on. The user-defined neighbourhood size determines the neighbourhood search perimeter in which thresholding occurs.

For example, employing a local neighbourhood density threshold within 7 × 7 neighbourhood matrix investigates all 8 first neighbour pixels as well as pixels along the horizontal, vertical and diagonals of the second, third and other neighbours are interrogated. If a user defined density of white pixels are found within the search perimeter, the central pixel is assigned as a white pixel. Alternatively, if a user defined density of white pixels are not found within the search perimeter, the central pixel is assigned as a black pixel. Fig. 2d shows the result of the application of a local neighbourhood threshold of 1 in a 3 × 3 neighbourhood matrix to the inverted binary map in Fig. 2c.

Following local neighbourhood density thresholding, a dilation kernel may or may not be applied around the central white pixel. During dilation, the binary value of each pixel is investigated. If the pixel in question has a binary value of 1 (*i.e.* the pixel is white), a dilation kernel is applied which expands the pixel size. For example, a dilation kernel of 3 means that a 3 × 3 matrix of 8 first neighbour pixels, as well as the central white pixel, are designated as white pixels. If the investigated pixel has a value of 0 (*i.e.* the pixel is black), no dilation occurs. Finally, the “Median Filter” script was applied to all combinations with a filter size of 3. Each pixel is filtered by taking the binary value of pixels in a surrounding 3 × 3 pixel matrix, sorting them in order and replacing its value with the median of pixel values (*i.e.* the central pixel). This acts to tidy up the edges and remove any remaining black pixels within white regions (Fig. 2e).

**Fig. 3:**
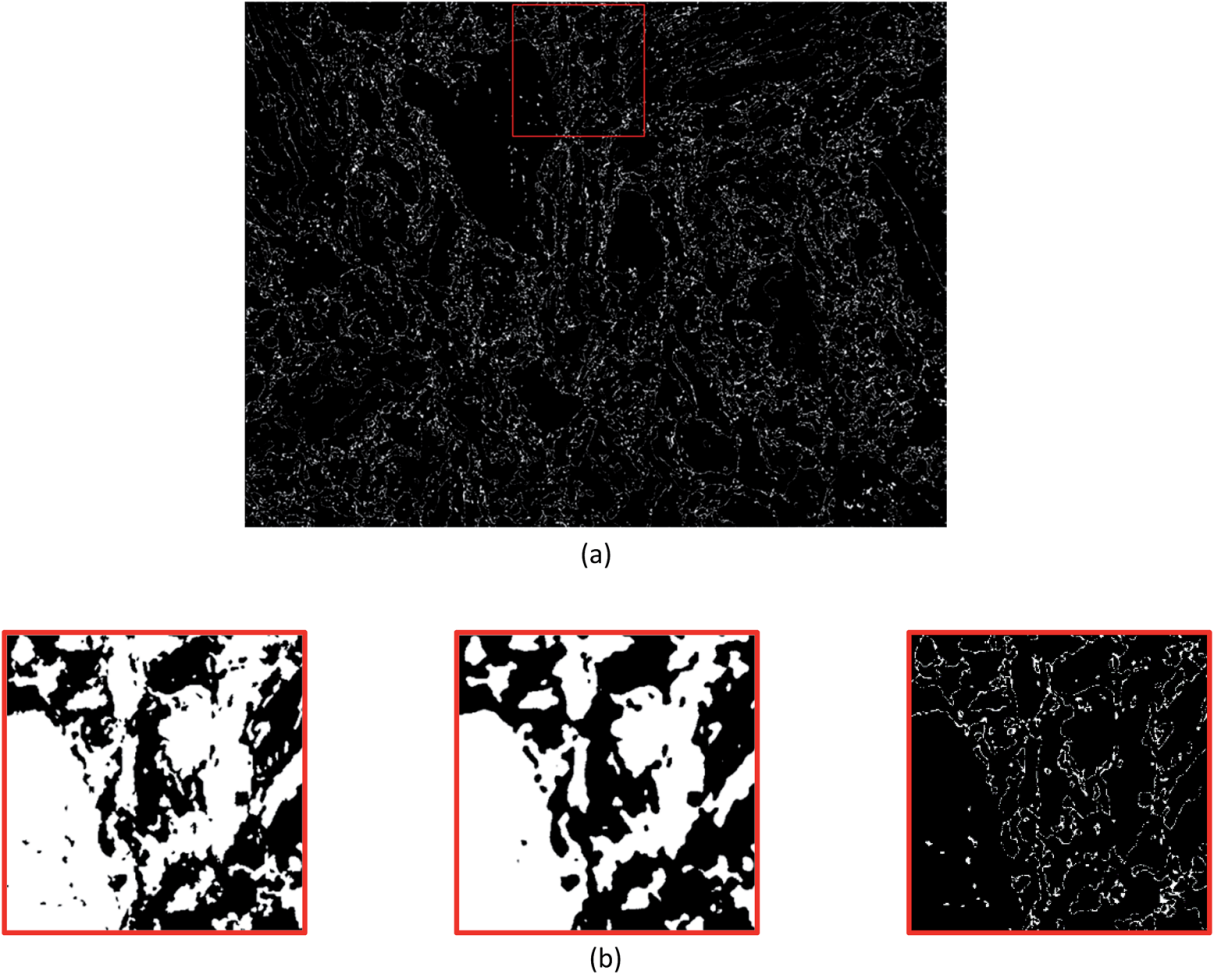
(a) The resultant absolute difference image obtained from Fig. 1d (the reference phase map) and Fig. 2e (the phase map from segmentation) on using the ‘Image Calculator’ function in ImageJ. (b) Zoomed-in views of regions highlighted in red in Fig. 1d (far left) and 2e (middle) that result in the absolute difference image (far right). The absolute difference image (far right) returns black pixels when the images on the far left and middle are equivalent and white pixels when they differ.

Since this study aims to assess the efficacy of segmentation, various combinations of neighbourhood matrix size, neighbourhood density threshold and dilation kernel size were applied (Table 1) to the absolute error in the phase map from segmentation (Table 1) was determined by calculating the absolute difference between this segmented map and the reference phase map obtained by combined EDS + EBSD (Fig. 1d) on a per-pixel basis using the ‘Image Calculator’ function in ImageJ. The absolute difference between Fig. 1d and 2e resulted in the image shown in Fig. 3a. The resultant image contains white pixels wherever a mismatch in binary pixels was detected in Fig. 1d and 2e. Consequently, the absolute error is defined as a percentage of the number of mismatched white pixels to the total number of pixels (Table 1).

**Table 1:** The various combinations of neighbourhood matrix size, neighbourhood density threshold and dilation kernel size applied to the sulphur-K map shown in Fig. 2c. The absolute error in the phase map obtained from segmentation of the sulphur-K map was determined by computing the absolute difference between it and the reference phase map obtained by combined EDS + EBSD (Fig. 1d) on a per-pixel basis

Neighbourhood matrix	Neighbourhood density threshold	Dilation kernel size	Absolute error (%)
3 × 3	1	0	5.3
		3	12.6
		5	20.6
	2	0	7.2
		3	7.5
	3	0	15.8
		3	5.7
5 × 5	1	0	6.4
		3	9.4
	2	0	14.1
		3	6.1
	3	0	31.2
		3	9.1
7 × 7	1	0	9.3
		3	8.1
	2	0	30.0
		3	7.7
	3	0	40.2
		3	15.6

## 3. Results and discussion

During conventional EDS mapping the electron beam rasters over an area of interest multiple times for a fixed time-period or a user-defined number of counts. On the other hand, during combined EDS + EBSD mapping the beam rasters over an area of interest only once for a given mean dwell time per pixel.

Given the high sample tilt, the associated interaction volume effects and the rather limited EDS-data acquisition conditions, the absolute elemental counts recorded by the phases during combined EDS + EBSD may be prone to error. However, the authors contend that despite the limitations imposed by combined EDS + EBSD mapping, when phases have similar crystal structures and lattice parameters, the relative variation in their elemental counts can be exploited to effectively discriminate them. Thus, given optimised EDS + EBSD acquisition parameters and unique phase chemistries, even a relatively low-density element (sulphur-K in the present study) can be used to successfully undertake phase discrimination during mapping (Fig. 1c).

### The efficacy of the segmentation methodology

The value of the absolute error associated with phase discrimination *via* the segmentation methodology is heavily dependent on the user-defined thresholds for grayscaling of the initial sulphur-K map (Fig. 2c), the neighbourhood matrix size, the neighbourhood density threshold and the dilation kernel size (Fig. 2d). The absolute errors associated with various combinations of thresholding parameters are listed in Table 1. It should be noted that although a range of grayscale thresholds were applied to this image, they are not reported here. All subsequent analyses and absolute error calculations are based on a grayscale threshold between 200 and 255, as this threshold returned the lowest absolute error following segmentation.

As shown in Table 1, larger absolute errors can be most commonly associated with large neighbourhood density thresholds, dilation kernel sizes and neighbourhood matrix sizes. The neighbourhood density threshold is particularly sensitive at interfaces of high and low sulphur counts (Fig. 2a); which in turn correspond to the Bi_2_Te_2_S and Bi_2_Te_3_ phases (Fig. 1c and 2f) as well as white and black pixels (Fig. 1d and 2e). In the interests of simplifying explanations, the following discussion refers to white and black pixels only.

**Fig. 4:**
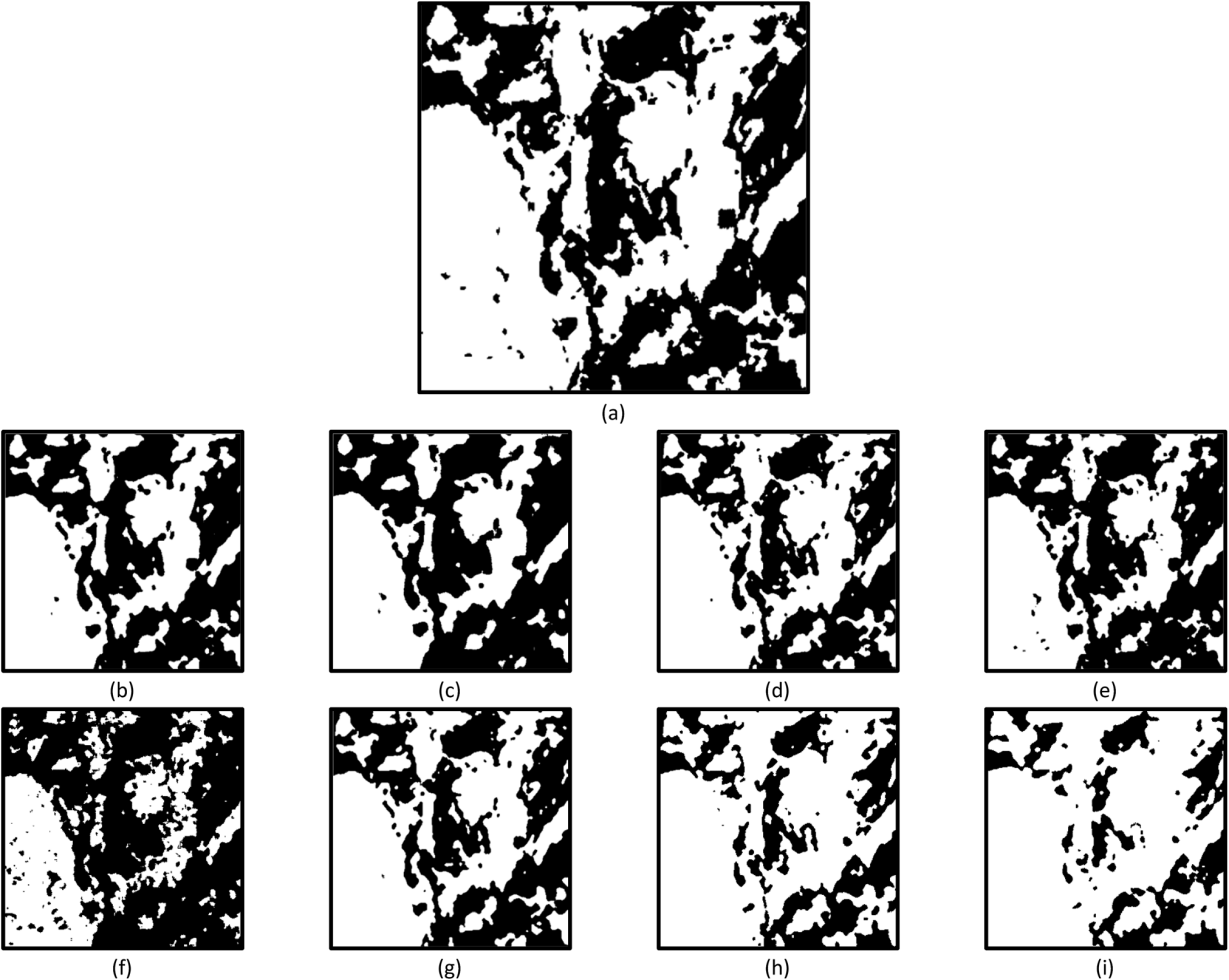
(a) A zoomed-in view of a region taken from the binarised reference phase map shown in Fig. 1d. (b, c) The equivalent region when a neighbourhood density threshold of 1, no dilation kernel, and a 5 × 5 and 7 × 7 neighbourhood matrix size are respectively used for phase segmentation. (d–f) The equivalent region when a 3 × 3 neighbourhood matrix size, no dilation kernel and a neighbourhood density threshold of 1, 2 and 3 are respectively used for phase segmentation. (g) The equivalent region when a neighbourhood density threshold of 3, a 3 × 3 neighbourhood matrix size and a dilation kernel size of 3 is used for phase segmentation. (h, i) The equivalent region when a neighbourhood density threshold of 1, a 3 × 3 neighbourhood matrix size and a dilation kernel size of 3 and 5 are respectively used for phase segmentation. A median of size 3 was applied to all maps shown.

Fig. 4a shows a zoomed-in view of a region of interest from the binarised reference phase map shown in Fig. 1d. The former will be used to discuss the implications of increasing the neighbourhood matrix sizes, neighbourhood density thresholds and dilation kernel sizes when segmenting for phase discrimination.

The use of neighbourhood matrix sizes larger than 3 × 3 appeared to have limited success; regardless of the associated neighbourhood density threshold and dilation kernel applied thereafter. For example, Fig. 4b and c are representative examples of the results obtained when a 5 × 5 and 7 × 7 neighbourhood matrix sizes are applied within which a neighbourhood density threshold of 1 was used. No kernel dilation was applied thereafter. It is evident that as the neighbourhood matrix size increases, contiguous white pixel regions shrink while approximately retaining their interfaces. The absolute error involved with larger neighbourhood matrix sizes often improved when coupled with neighbourhood density thresholds greater than 2 (Table 1). However, in this case, the interfaces are compromised. This is a result of the interface being reconstructed from excessive growth of contiguous white regions when a large neighbourhood matrix is employed, followed by shrinkage of these regions when a neighbourhood density threshold is applied.

It is pointed out that larger neighbourhood density thresholding can still result in reduced absolute errors of ∼≤10% when a dilation kernel is used thereafter (Table 1). For instance, if the results obtained when a 3 × 3 neighbourhood matrix size within which a neighbourhood density threshold of 3 is applied, followed by dilation kernel size of 3, the absolute error improves by ∼10% (compare absolute errors of 15.8% and 5.7% in Table 1). However, it must be understood that while the absolute error is reduced, the integrity of interfaces is often severely compromised (Fig. 4g) – the structure is once again convoluted during reconstruction. In this case, it is the responsibility of the user to determine whether large neighbourhood density thresholding followed by application of a dilation kernel is suitable for the data set in question.

Fig. 4h and i are representative examples of the results obtained when a 3 × 3 neighbourhood matrix size within which a neighbourhood density threshold of 1 is applied, followed by dilation kernel sizes of 3 and 5, respectively. Compared to Fig. 4a, the application of a small neighbourhood density threshold coupled with larger dilation kernel sizes greatly exaggerates the size of coarse contiguous, white pixel regions by promoting the coalescence and incorporation of small groups of white pixels into them (Fig. 4h and i).

Table 1 shows that the application of dilation kernels can result in reduced absolute errors when neighbourhood density thresholds of 2 or higher are applied. As discussed previously, the application of large neighbourhood density thresholds can erroneously reduce the fraction of white pixels while severely compromising interfaces. Thereafter, the use of large dilation kernel sizes merely serves to compensate for the loss of white pixels from the previous neighbourhood density threshold step while retaining the erroneous interfaces. It was deemed unnecessary to report the absolute error associated with the application of a dilation kernel size of 5 for the majority of cases due to its tendency to visibly and severely compromise the boundary interface between the two phases.

For all combinations of parameters shown in Table 1, the application of a median filter of size 3 constituted the final step of the segmentation methodology. If a median filter were not applied, an erroneously large density of black pixels within white regions or *vice versa* would remain in the segmented image. The application of median filters greater than 3 were also tested, and disregarded due to the elimination of an erroneously high fraction of fine white pixel regions. A comparison of Fig. 2d and e highlights the necessity of applying a median filter; such that a filter size 3 effectively removes artefacts and smooths interfaces. It should also be noted that while median filtering may have the adverse effect of reducing or eliminating small pixel groups, the overall accuracy of the segmented image is improved following median filtering. For example, prior to the application of a median filter, the segmented phase map (Fig. 2d) had an absolute error of 7.45% relative to the reference phase map. Upon applying a median filter of size 3, the absolute error reduced to 5.3%.

The above assessment of the parameters employed during the segmentation methodology suggests that user discretion is required to ensure the smallest absolute error between the segmented and reference phase maps. However, for an optimised combination of segmentation parameters (as detailed in Section 3), an absolute error of ∼5% between the segmented and reference phase maps can be obtained. It must be noted that an understanding of how each parameter in the segmentation methodology affects the absolute error is essential to employing the methodology appropriately. Direct observation and comparison of interfaces from segmented and reference maps must be made to conclude the quality of the segmented map.

Error is most commonly localised in the 2–4 pixel region at the boundary interface between phases. In the context of this study, the error is detrimental for fine sulphur-rich regions and is in part, attributed to the large area of the EBSD map and the employed step size. In cases where interface integrity is particularly important, EBSD maps should be acquired using lower accelerating voltage and probe current at higher magnification and even smaller step sizes. It follows that such an approach would be a suitable means to reduce the absolute error associated with phase segmentation even further. This result, and the strategy to further reduce the absolute error, demonstrates the suitability of the segmentation methodology as a means to discriminate phases *via* the post-processing of elemental maps.

## Conclusions

The efficacy of the proposed segmentation methodology *via* post-processing the image of an elemental map for phase discrimination was critically assessed. Reference EDS spectra of Bi_2_Te_3_ and Bi_2_Te_2_S in the multiphase Bi_2_Te_2.5_S_0.5_ compound were distinct enough to discriminate the two during map acquisition. When the segmentation methodology is optimised, the segmented phase map returns a minimum absolute error of ∼5% compared to the reference phase map after map acquisition.

## Conflicts of interest

There are no conflicts to declare.

## Supplementary Material
